# A Treatise on the Practice of Medicine

**Published:** 1861-08

**Authors:** 


### Book Notices.

A Treatise on the Practice of Medicine.—By Edwin R. Maxon, M. D., formerly Lecturer on Institutes and Practice of Medicine in the Geneva Medical College. Philadelphia: Lindsay & Blackiston. 1861. 8vo. pp. 705.

It is to be regretted that his "exciting and extensive practice," during two years of which ex-Prof. Maxon wrote his *Treatise*, was not of so engrossing a nature as to preclude the, no doubt worthy, practitioner from essaying the *role* of author. That it was engrossing enough to prevent the writer fulfilling his intentions, a perusal amply satisfies us; for it is neither "a valuable and interesting work for the student," nor "a reference for practitioners of medicine generally," as the author sets forth in the preface. But, surely, no pressure of practice could be so great as to crowd out of his mind, who aspires to rank with Watson, Wood and Eberle, all observance of Lindley Murray, all semblance of regard for the genius, construction, and resources of the language in which he writes. The peculiar, offensive character of comparisons is well known; and yet such an effort as this, cannot fail to provoke them, when we remember the graceful ease of eloquence, the manly, sensible modesty, the depth of research, and the wealth of wisdom, skill and learning which conspire to make Watson's *Practice* the model text-book for the student, the fascinating companion of the practitioner.

Dogmatic in tone, obscure and shallow in treatment, inelegant and monotonous in style, in this first, we cannot look forward with much zest to the second, edition of the *Treatise*, which its sanguine author already anticipates; but charitably consigning it to a merciful repose in the dusty fastnesses of our upper shelves, counsel its progenitor to devote himself to the duties and demands of his practice, as one to whom the *mare nigrum* offers only an inglorious grave, instead of bearing him, on its inky waves, to the renown and glory of successful authorship.

Our Exchanges.—With the exception of some half-dozen of the leading ones, the thirty or forty-odd periodicals which were wont to flood our sanctum, month after month, are now *non est*,—whether practically or figuratively declared "contraband of war," we know not; nor whether the fault be due to the postal arrangements,—or derangements. The sterling *Lancet*, and the portly *Braithwaite*, the dignified *American Journal* and the scholarly *Medico-Chirurgical Review;* with the elegant *American Medical Monthly*, and the *Berkshire*, of the quarterlies and monthlies for July, are received, as also the more sprightly weeklies of Boston, New York and Philadelphia. But our cotemporaries of Cleveland, Columbus, Cincinnati, New Lebanon, St. Louis, Louisville, and those not mentioned in the above list, of the leading eastern cities, have, as yet, at the close of the month, failed to put in an appearance. Of course, those of our unfortunate Southern brethren of Secessia, have sadly-weighty reasons for their absence.

